# Hierarchical Nanoflowers on Nanograss Structure for a Non-wettable Surface and a SERS Substrate

**DOI:** 10.1186/s11671-015-1214-7

**Published:** 2015-12-30

**Authors:** Jun-Young Lee, Jaehyun Han, Jihye Lee, Seungmuk Ji, Jong-Souk Yeo

**Affiliations:** School of Integrated Technology, Yonsei University, Incheon, Republic of Korea; Yonsei Institute of Convergence Technology, Yonsei University, Incheon, Republic of Korea

**Keywords:** CuO nanostructure, Hierarchical structure, Superhydrophobic, Oleophobic, Non-wetting, Surface-enhanced Raman spectroscopy

## Abstract

Hierarchical nanostructures of CuO nanoflowers on nanograss were investigated for self-cleaning and surface plasmonic applications. We achieved the hierarchical nanostructures using one-step oxidation process by controlling the formation of flower-like nanoscale residues (nanoflowers) on CuO nanograss. While the nanograss structure of CuO has a sufficient roughness for superhydrophobic characteristics, the additional hierarchy of nanoflowers on nanograss leads to a semi-reentrant structure with a high repellency even for a very small droplet (10 nL) of low surface tension liquid such as 25 % ethanol (~35 mN/m), thus providing non-wettable and self-cleaning properties. Furthermore, the CuO hierarchical nanostructure serves as a substrate for surface-enhanced Raman spectroscopy (SERS). Both of the CuO nanograss and nanoflower provide many nanoscale gaps that act as hot-spots for surface-enhanced Raman signal of 4-mercaptopyridine (4-Mpy), thus enabling a non-destructive detection in a short analysis time with relatively simple preparation of sample. Especially, the CuO nanoflower has larger number of hot-spots at the nanogaps from floral leaf-like structures, thus leading to three times higher Raman intensity than the CuO nanograss. These multifunctional results potentially provide a path toward cost-effective fabrication of a non-wettable surface for self-maintenance applications and a SERS substrate for sensing applications.

**Electronic supplementary material:**

The online version of this article (doi:10.1186/s11671-015-1214-7) contains supplementary material, which is available to authorized users.

## Background

Nanoscale architectures have attracted strong interests due to their unique properties from large surface-to-volume ratio, nano-roughness, and nanoscale gap formations. These properties are essential to enable specific surface functionalities such as liquid super-repellence and nanoscale plasmonic enhancement. The aforementioned functionalities are imperative for durable surface protections and high-efficiency bio-optical sensing utilizing surface-enhanced Raman spectroscopy (SERS).

As one of the nanoscale architectures, copper oxide nanostructures offer unique characteristics and advantages. First, Cu is one of the most commonly used materials in numerous commercial products. Also, Cu oxidation and their nanostructures have been widely studied for a long time. Second, CuO nanostructures are easy to synthesize via cost-effective bottom-up process such as chemical vapor deposition (CVD) [1], thermal oxidation in air [2–4], and hydrothermal methods [5, 6]. For example, various Cu-related oxide nanostructures such as CuO nanowire, Cu_2_O nanotube, Cu_2_O nanoribbon, and CuO plates can be made using a simple oxidation technique [5, 7]. Third, CuO is a chemically stable material as a base for hierarchical structures [8–10] or for functionalized structures [11] in various applications [8–10, 12]. As the surfaces based on CuO nanostructures also provide extremely high roughness, they are suitable for self-cleaning [8, 9], anti-condensation [11], and anti-corrosion [13] applications. Especially, superamphiphobic surfaces that can repel water as well as low surface tension liquids were investigated using CuO and hydrophobic wax-based hierarchical structures [8, 9].

While CuO nanostructures provide many advantages as mentioned above, it is still desirable to develop three-dimensional (3D) hierarchical nanostructures of CuO for improved and reliable non-wetting property with the increased surface roughness or for effective surface-enhanced Raman detection of molecules by the formation of nanoscale gaps [14–19].

**Fig. 1:**
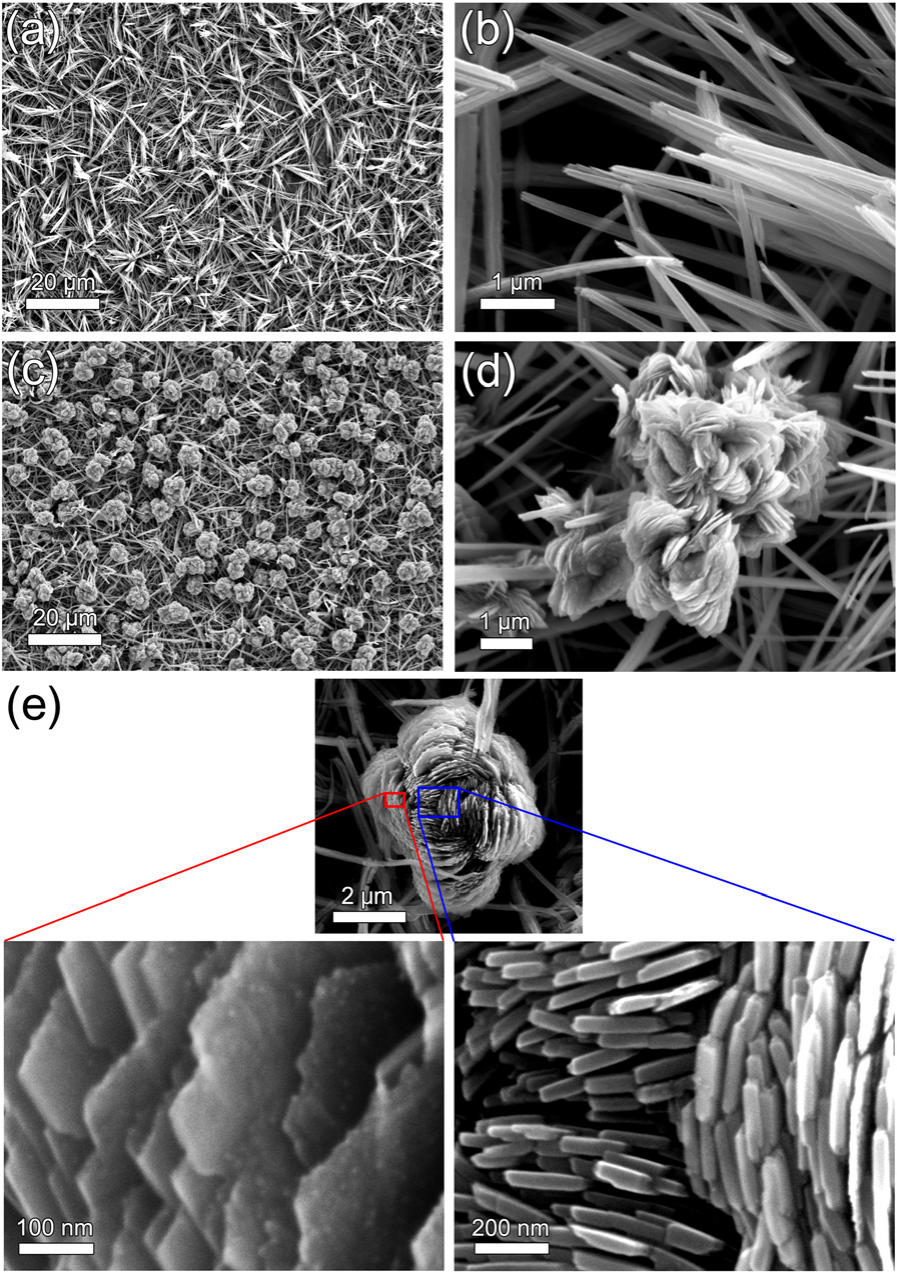
CuO nanoflowers on nanograss SEM images of CuO surface covered by **a**, **b** CuO nanograss and **c**, **d** hierarchical structure of CuO nanoflowers on nanograss. **e** High-resolution SEM images of CuO nanoflowers

In this work, we demonstrate the 3D CuO hierarchical nanostructures consisting of 1D nanograss and flower-like 3D nanostructures (nanoflowers) grown at once via a single-step oxidation process (Fig. 1). Our CuO hierarchical nanostructures have a robust repellency for low tension liquid with 35 mN/m as well as water due to the semi-reentrant structure with the nanoflower on nanograss geometry. Furthermore, the floral geometry in the CuO hierarchical nanostructures provides abundant nanoscale gaps. As a result, our CuO hierarchical nanostructures show three times higher enhancement of SERS signal for 4-mercaptopyridine(4-Mpy) compared to the nanograss geometry alone.

## Methods

### Fabrication of CuO Nanoflowers on Nanograss

The fabrication process of CuO nanoflowers on nanograss is divided into two steps. First, Cu foil was oxidized in an ammonium ambient solution of 2.5 M NaOH and 0.1 M (NH_4_)_2_S_2_O_8_ at 4 °C for 10, 30, and 60 min [8, 9, 11, 20, 21]. In this step, the generated nanostructures contain Cu(OH)_2_ phase dominantly. Second, the samples were dehydrated at 180 °C for 60 min to transform the Cu(OH)_2_ to CuO phase [20, 21]. Hierarchical formation of the CuO nanoflowers among the CuO nanograss was controlled during the final cleaning process. The more details on the mechanism of forming CuO nanoflowers are discussed in the “Results and Discussion” section of this paper.

### Characterization of CuO Nanostructures

Surface morphologies of the fabricated CuO nanograss and CuO nanoflowers were characterized using field emission scanning electron microscopy (FE-SEM) (JSM-7100F, JEOL). To confirm the crystallinity of CuO nanostructures, X-ray diffraction analysis (XRD) (Smartlab X-ray Diffractometer, Rigaku) was conducted. High-resolution analysis of as-fabricated CuO nanostructures was conducted using the aberration (C_s_) corrected scanning transmission electron microscopy (JEOL JEM-ARM200F 200 kV FEG-STEM/TEM) equipped with a C_s_ corrector (CEOS) on the illumination system.

### Non-wetting Coating on CuO Hierarchical Nanostructures

To make non-wettable surfaces, the surface of CuO nanostructures was chemically modified by a self-assembled monolayer (SAM) of trichloro(1H,1H,2H,2H-perfluorooctyl)silane (FOTS) in a vacuum desiccator for 2 h. The wettability of FOTS-coated CuO nanostructures was investigated using a contact angle (CA) goniometer (Dataphysics, OCA15EC) with ultrapure DI water (72.2 mN/m), glycerol (63.4 mN/m), ethylene glycol (47.3 mN/m), olive oil (32 mN/m), and 8~55 % ethanol (50~27 mN/m) [22] at room temperature. CA hysteresis (CAH) for each liquid was also measured by continuous liquid injection and retraction using a microsyringe (Hamilton) and a microneedle (inner diameter of <0.1 mm).

### Fabrication of SERS Substrate

Ag was deposited on the CuO nanostructures with various thicknesses from 10 to 80 nm using a thermal evaporator. Then, the uncoated and Ag-coated CuO nanostructures were immersed in a 0.001 M methanol-based 4-Mpy for 15 min in order to evaluate SERS enhancement.

Raman shift and SERS enhancement were observed using a Raman spectrometer (Horiba, LabRAM ARAMIS Spectrometer). Excitation of 532-nm wavelength was used with 50 mW. The laser beam was focused with 500 nm in size and the spectra were collected for 10 s.

## Results and Discussion

### Hierarchy of CuO Nanoflowers on Nanograss

As-grown CuO nanowires look like grass, randomly aligned as shown in Fig. 1a, b. CuO nanoflowers are placed on CuO nanograss matrix instead of directly contacting the bottom surface of Cu foil, thus forming a hierarchical structure as seen in Fig. 1c, d. The average thickness of CuO nanoflowers’ leaves is around 70 nm similar to the diameter of nanograss as shown in Fig. 1d. The leaves of CuO nanoflower (plate-like CuO crystal) are densely packaged with narrow nanoscale gaps (nanogaps) in between the nanoscale leaves. The growth mechanism of CuO nanowire has been well explained in the previous reports [8, 9, 11, 20, 21]. However, the formation of CuO nanoflowers on CuO nanograss matrix requires more concrete understanding for the controlled fabrication of such nanostructures.

**Fig. 2:**
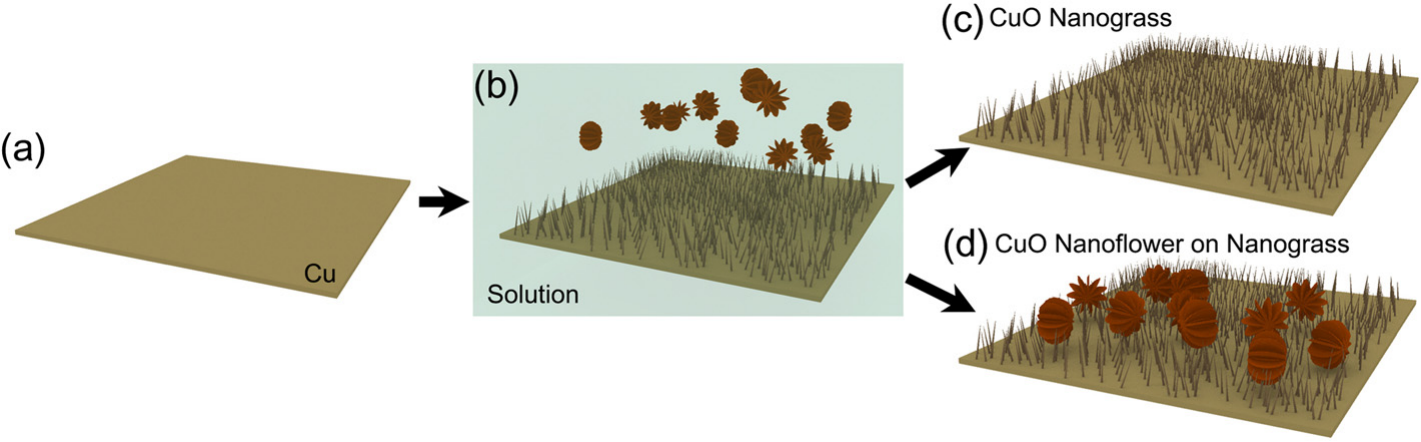
Formation of CuO nanostructures. Schematic images of hierarchical CuO flower-like structure, **a** Cu substrate, **b** CuO nanograss fabrication in a solution and flower-like floating structures, **c** 1D CuO nanograss surface, and **d** 3D CuO nanoflowers on nanograss structure

The oxidation process in ammonium ambient solution generates various nanostructures such as nanosheets, nanobelts, nanoplates, as well as nanograss simultaneously [6]. During the oxidation process on Cu foil, Cu transitions to Cu(OH)_2_ from the reaction with part of Cu surface. Some parts of Cu surface can react faster than the other part leading to the nonuniform oxidation and the corresponding formation of nanostructures. This difference in the oxidation speed can render some nanostructures chipped off from the Cu foil substrate. In order to minimize surface energy, some of these nanostructures can agglomerate together to form flower-like clusters as illustrated in Fig. 2b [6]. These chipped off Cu(OH)_2_ nanostructures float in the solution changing the color of the solution from colorless to blue.

Whether to form nanograss only or nanoflowers on nanograss depends on the level of cleaning in the subsequent cleaning process. With thorough cleaning, only Cu(OH)_2_ nanograss remains on Cu substrate (Fig. 2c). On the other hand, gentle cleaning of the surface enables the formation of the hierarchical Cu(OH)_2_ nanoflowers on nanograss matrix (Fig. 2d). After the dehydration process, the agglomerated flower-like clusters are tightly bonded down to the CuO nanograss. Therefore, the number of CuO nanoflowers can be controlled by varying the level of cleaning in the final process.

**Fig. 3:**
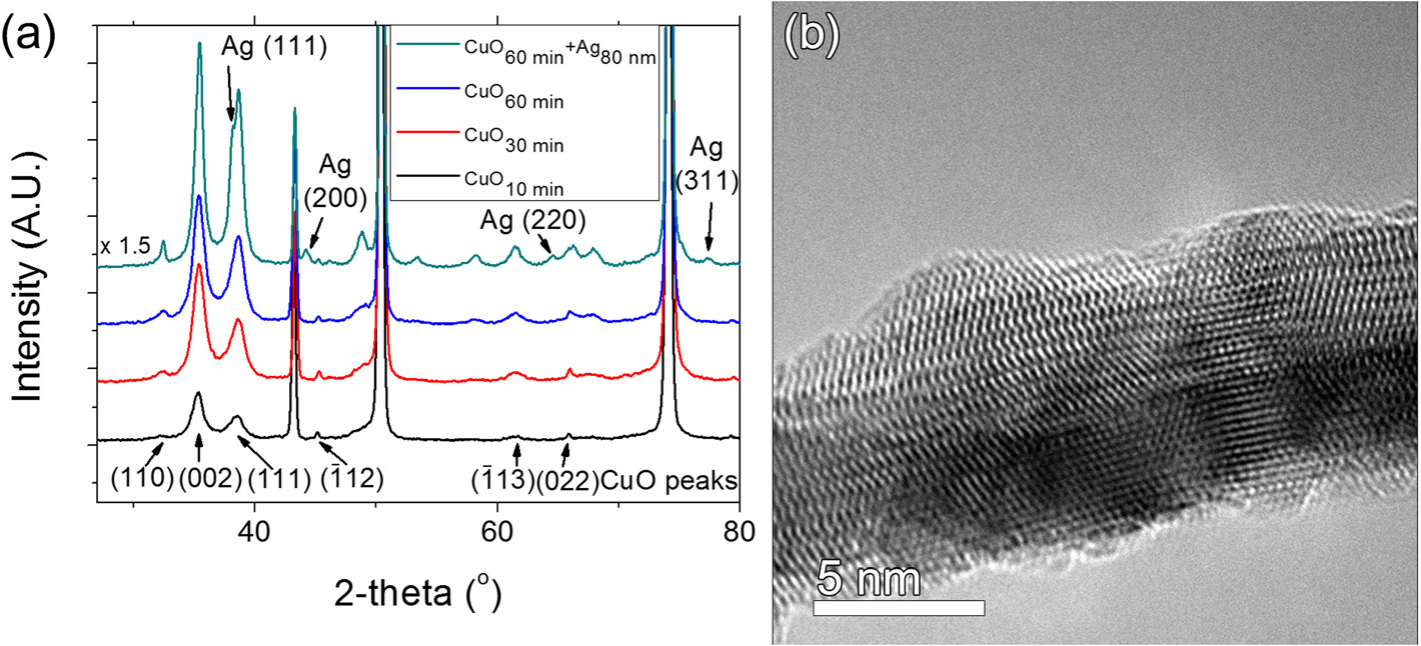
Characterization of CuO nanostructures. **a** XRD analysis of as-grown CuO nanostructures as a function of oxidation time and a CuO surface after silver layer formation. **b** A HRTEM image of a CuO nanograss

XRD analysis reveals that the nanostructures consist of polycrystalline CuO. Peaks of monoclinic CuO (110), (002), (111), $\left(\overline{1}12\right)$, $\left(\overline{1}13\right)$, and (022) plane are detected at 32.5°, 35.8°, 38.2°, 45.2°, 61.5°, and 65.9°, respectively, indicating a rather ordered formation for the CuO nanostructures (The Joint Committee on Powder Diffraction Standards (JCPDS) Card No. 41-0254). Figure 3a shows that the intensity of CuO crystal increases with oxidation time as the surface is covered more with crystalline structures. Peaks of Ag (111), (200), (220), and (311) planes are detected at 37.93°, 44.14°, 64.68°, and 77.55°, respectively, indicating the formation of Ag layer on CuO nanostructures (JCPDS Card No. 00-001-1164). The crystallinity of a CuO nanowire can also be confirmed by high-resolution transmission electron microscopy (HRTEM) image as shown in Fig. 3b.

### Non-wettable Surface Using Hierarchical CuO Nanostructures

Hierarchical CuO nanostructures of nanoflowers on nanograss have roughness sufficiently large enough to obtain superhydrophobic characteristic. Since CuO material itself is hydrophilic, the surface of the hierarchical nanostructures is coated with FOTS in order to obtain non-wetting property. Self-assembled FOTS monolayers reduce the surface energy of the nanostructures. When the water droplets are placed on the nanostructured surface, the functionalized surface exhibits a Cassie wetting state by trapping the air between the structures and beneath the liquid. This phenomenon is explained by the well-known theory of non-wettable hierarchical surface based on the Cassie-Baxter model [23–26] as follows:

$$ \cos {\theta}_n^{*}=\left(1-{f}_{\mathrm{LV},n}\right) \cos {\theta}_{n-1}^{*}-{f}_{\mathrm{LV},{n}^{*}} $$

Here, *f*_LV_ is the area fraction of the liquid-vapor (air) interface and *f*_LV,*n*_ is the area fraction of the liquid-vapor (air) interface for *n*th level of structures [26]. Hierarchical nanostructures have relatively larger area fraction of the liquid-vapor interface than unary nanostructures, thus providing better non-wetting property according to the theory above. As CuO nanoflowers provide additional roughness on CuO nanograss surface, the surface with hierarchical nanostructures has shown superhydrophobic characteristic.

The water CA of CuO hierarchical nanostructures is >160° and the CAH is <1° (using a 7-μL droplet of ultrapure DI water). The CA and CAH indicate self-cleaning property on the nanostructured surface where dusts are removed as the water droplet rolls off the surface. Liquid repellency is also affected by the nanoflower structure. As the nanoflower structure provides semi-reentrant curvature on the surface, the hierarchical surfaces can repel not only DI water but also various low surface tension liquids such as glycerol, ethylene glycol, olive oil, and diluted ethanol (mixed with various amounts of water).

**Fig. 4:**
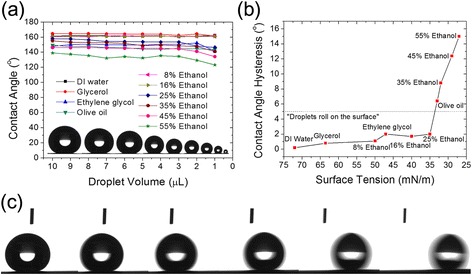
Wetting property of hierarchical surface with CuO nanoflowers for various liquids. **a** The change of contact angles of various liquids as a function of droplet volume using dropping test. **b** The average contact angle hysteresis of various liquids as a function of surface tension (droplets of liquids have small CAH of <5° roll on the surface). **c** Continuous optical images of 7 μL, 25 % ethanol droplet which is rolling on the nanoflower applied hierarchical CuO surface (the total rolling time is 203 ms)

Structural density in the hierarchy provides additional functionality of non-wetting characteristic with regard to a droplet volume. In order to investigate this effect, we have measured contact angles at various droplet volumes using different low surface tension liquids. The hierarchical nanostructures with higher density of nanoflowers can withstand the increase in Laplace pressure, thus maintaining superhydrophobic and oleophobic characteristics even for a small droplet (Fig. 4 and Table 1). The hierarchical nanostructures with nanoflowers exhibit oleophobic characteristics with high contact angle of >140° and low contact angle hysteresis <10° with 7-μL droplets of glycerol, ethylene glycol, olive oil, 8 % ethanol, 15 % ethanol, 25 % ethanol, 35 % ethanol, and 45 % ethanol. Especially, for the droplets of glycerol, ethylene glycol, 8 % ethanol, 15 % ethanol, and 25 % ethanol, the surface with nanoflowers has shown super-phobic characteristic (extremely high contact angle of >150° and low CAH of <5°). Figure 4c shows an example of this super-phobicity with the continuous images of 25 % ethanol droplet rolling on the FOTS-coated hierarchical nanostructures. These non-wetting properties are maintained well with a relatively small droplet volume of 1 μL. The hierarchically nanostructured surface shows high contact angle >140° for a 1-μL droplet liquid with surface tension ranging above 33 mN/m as shown in Fig. 4a. These superhydrophobic and oleophobic characteristics for a small droplet volume demonstrate the non-wettable properties of the surface with hierarchical nanoflowers on nanograss.

**Table 1:** Wettability of the hierarchical nanoflower on nanograss surface

Liquids	Surface tension (mN/m)	CA (°) 7 μL droplet	CA (°) 1 μL droplet	CAH (°)
DI water	72.2	161.9	162.4	0.2
Glycerol	63.6	164.4	161.7	0.8
Ethylene glycol	47.1	150.1	141.6	2
Olive oil	32	146.2	143.7	8.8
8 % ethanol	50	161.7	160.7	1.1
16 % ethanol	40	161.2	161.1	1.7
25 % ethanol	35	155.8	154.6	2
35 % ethanol	33	152.7	141.1	6.4
45 % ethanol	29	145.9	134.2	12.4
55 % ethanol	27	132.2	123.2	15

Averaged CAs and CAHs of various liquids on hierarchical nanoflowers on nanograss structures

**Fig. 5:**
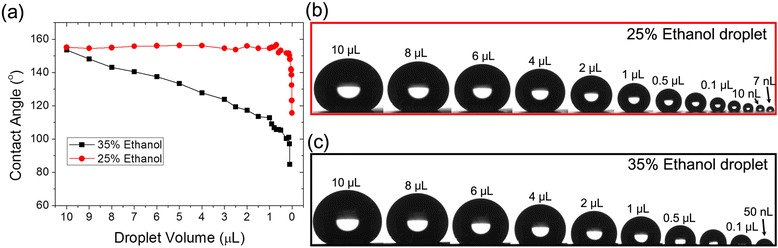
Evaporation test. **a** The change of contact angles with 25 % ethanol and 35 % ethanol as a function of droplet volume using evaporation of liquids, and the relevant optical images of **b** 25 % ethanol and **c** 35 % ethanol droplets

In addition, the evaporation of the droplet on the surface allows us to evaluate how wetting changes as droplets get smaller and how droplets behave depending on whether they are pinned on the surface structures. According to the results of evaporation test as seen in Fig. 5, the surface shows a repellency for 25 % ethanol with the droplet volume as small as 10 nL. Since 25 % ethanol droplet is not pinned on the surface that has low CAH, decreasing volume of evaporating droplet also reduces the contact area between liquid and surface so the high contact angle is maintained around 150°. The liquid phobicity with contact angle above 90° is maintained for the droplet volume as small as 7 nL. In contrast, 35 % ethanol droplet (33 mN/m) is pinned on the surface that has higher CAH (>6°) than 25 % ethanol. Therefore, the contact area between liquid and surface is maintained while the droplet volume is reduced, thus resulting in the decrease of contact angle with evaporation as shown in Fig. 5c. For the 25 % ethanol that is not pinned on the surface, the results of contact angle measured both from dropping and evaporation tests are the same while the contact angle results are quite different for the 35 % ethanol that is pinned on the surface (Figs. 4a and 5a). All of these results are compiled in a separate video file as Additional file 1.

The enhanced non-wettable surface composed of the hierarchical nanostructures can be fabricated during one-step oxidation process without additional deposition steps. Therefore, this one-step fabrication of 3D hierarchy of nanoflowers on nanograss can be a low-cost approach to achieve robust functional surfaces.

### Raman Spectroscopy of 4-Mpy/Ag/CuO Nanostructures

The hierarchical surface is covered by dense nanograss and nanoflowers with many nanoscale gaps among the nanostructures. Taking advantage of such nanoscale gaps can lead to the enhancement of surface Raman signals. In order to make SERS substrate from the hierarchical CuO nanostructures, an Ag layer is formed on CuO nanograss and nanoflowers via thermal evaporation. Silver has a unique optical property compared to the other noble metals. It has the highest quality factors with the strong plasmon resonance ranging from 300 to 1200 nm in its spectrum. Due to the generation of strong plasmon at desired and wide spectral range, silver is a good material for making a qualified SERS substrate [27]. The thickness of silver layer is controlled from 20 to 80 nm. To reveal the feasibility of CuO nanostructures as a SERS substrate, the surface is tested with 4-Mpy as a probe molecule for the measurement of inelastic scattering by the surface-enhanced Raman spectroscopy. The surfaces are immersed in 0.001 M 4-Mpy solution for 15 min. Raman shifts from Ag/CuO nanostructures applied with various 4-Mpy are measured as a function of Ag layer thickness.

**Fig. 6:**
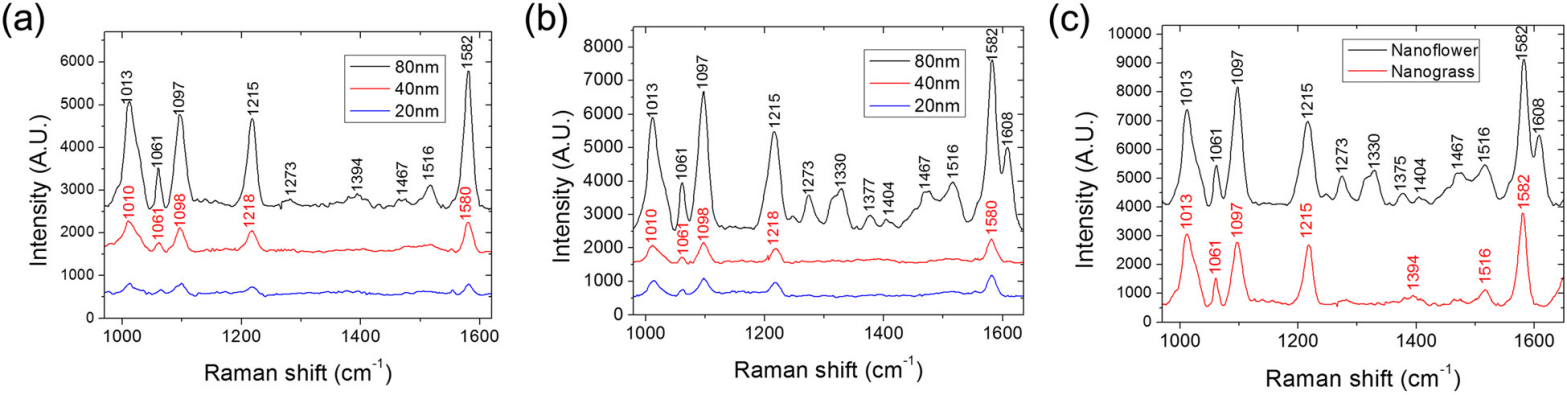
Raman spectroscopy of 4-Mpy/Ag/CuO nanostructures with various thicknesses of Ag layers deposited on **a** CuO nanograss and **b** CuO nanoflower. **c** Comparison of Raman spectroscopy data between nanograss and nanoflower coated with 80-nm-thick Ag layer

**Fig. 7:**
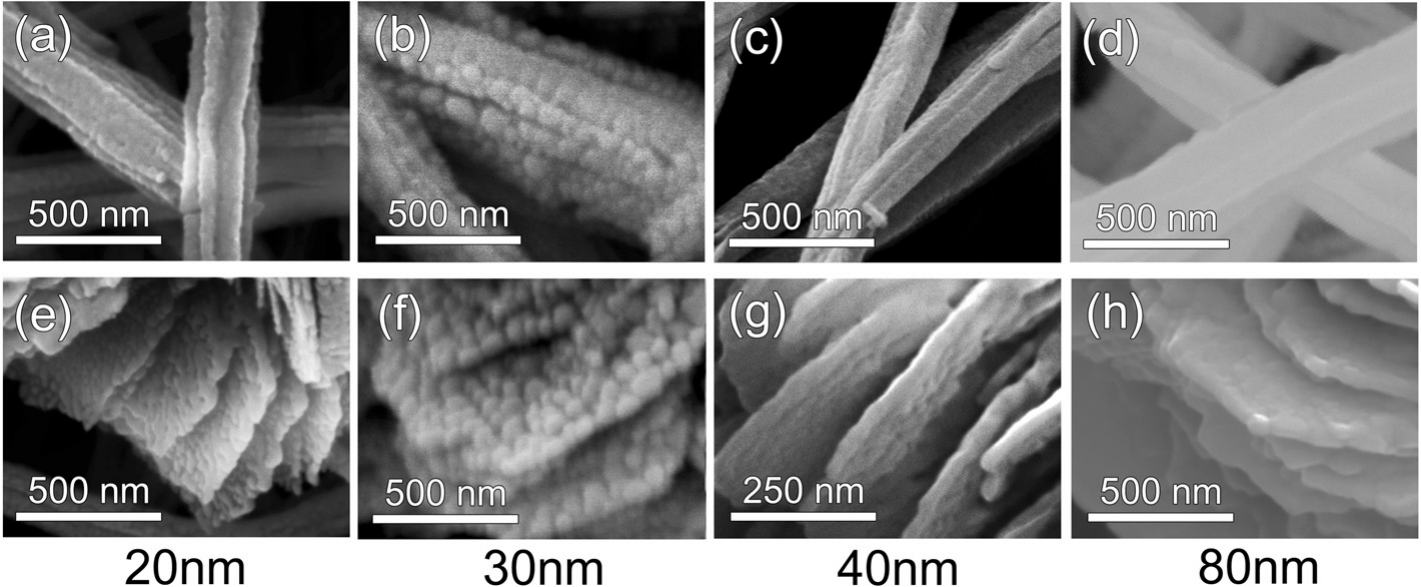
SEM images of Ag/CuO surfaces SEM images of **a**–**d** CuO nanograss and **e**–**h** CuO nanoflowers coated with Ag using various thicknesses of 20 nm (**a**, **e**), 30 nm (**b**, **f**), 40 nm (**c**, **g**), and 80 nm (**d**, **h**)

The SERS enhancement occurs by placing the Raman active molecules of 4-Mpy within the localized electric field crowding at the nanoscale gap formed of the deposited Ag layer on the roughened CuO surface. Figure 6 shows the Raman intensity and spectral shift for nanograss and nanoflower with varying thicknesses of Ag layer. The size of the probe beam in Raman spectroscopic measurement is adjusted to 2 μm so that the Raman intensity can be compared between the nanograss and the nanoflower. For CuO nanoflower with 80-nm Ag layer (Fig. 6b), the spectra exhibit a distinctive SERS enhancement compared to the CuO nanograss (Fig. 6a). As shown in Fig. 7, SEM images indicate the formation of the nanoscale gaps among the branches of the nanograss and the leaves of the nanoflowers, thus creating high density of hot-spots with excited on-resonant mode from such nanostructures.

For the cases of 20- and 40-nm-thick Ag layer samples, Raman shifts of 4-Mpy are observed at 1010, 1098, 1218, and 1580 cm^−1^. These peaks corresponding to Raman shifts stem from the interactions between Ag and 4-Mpy [28, 29]. On the other hand, the 80-nm-thick Ag layer samples have different Raman shift peaks such as 1013, 1061, 1097, 1215, 1273, 1330, 1377, 1404, 1467, 1516, 1582, and 1608 cm^−1^ as shown in Fig. 6b. The Raman shift peaks dominantly originate from the interactions of 4-Mpy situated among the Ag leaves [28, 30] as shown in Table 2. There are no Raman shift peaks from 1273 to 1516 cm^−1^ in Table 2 for the CuO nanoflower coated with 20-nm-thin Ag compared to the 80-nm-thick Ag.

**Table 2:** Raman spectroscopy

CuO nanoflower Ag 80 nm	CuO nanoflower Ag 20 nm	Ag mirror [30]	4-Mpy solid [36, 37]	CuO [28]	Assignment [36–38]
1013	1010	1010	989		Ring breathing
			1044	1022	β(CH)
1061	1061	1061	1080	1059	β(CH)
1097	1098	1097	1106	1106	Ring breathing/C-S
1215	1218	1219	1200	1208	β(CH)/(NH)
			1249		β(CH)
1273, 1330		1271	1288		β(CH)
1377, 1404			1394		ν(CC)
1467			1457		ν(C=C/C=N)
1516			1478		ν(C=C/C=N)
1582	1580	1580	1604	1584	ν(CC)
1608		1612	1612		ν(CC)

Raman shifts (cm^−1^) and assignments for 4-Mpy

For the condition of 20 to 40 nm thin layer deposition shown in Fig. 7a–c and e–g showing nanowires and nanoflower, Ag starts to nucleate with 20 nm condition and grows as islands with 30 to 40 nm condition on the surface providing weak Raman interaction with 4-Mpy molecules. Since the islands or sponge-like Ag aggregation provide low uniformity in shape and morphology, they form various resonant modes for a wide range of Raman peaks with low intensities, thus contributing weakly to SERS [31]. So the 20-nm-thin Ag on CuO nanostructures shown in Fig. 7a, e is not sufficiently meeting the condition to enable a strong finger print of the 4-Mpy molecules, thus acting as a non-SERS-active substrate [32].

Compared with the <40 nm deposition of silver, the 80-nm-thick Ag layer deposition leads to the continuous and uniform layer formation on the CuO-roughened surface, thus closing the nanogap further among the branches of the nanograss and the leaves of the nanoflowers as shown in Fig. 7d, h. This leads to the enhanced SERS signal enabling sensitive detection of the 4-Mpy molecules with the Raman peaks of 1273, 1377, 1404, 1467, and 1516 cm^−1^. These stably and uniformly made nanogaps now allow the CuO nanostructure to act as a SERS-active substrate.

In conclusion, the triggering factors for enhancement of surface Raman signal for detecting the target probing molecules greatly depend on a number of nanogaps, effective geometries, topologically active roughness, and suitable material conditions.

Considering these factors, we have used the CuO nanoflowers with their leaves made of crystal plates and coated with the silver layer to gain the Raman enhancement of molecules. The average enhancement of Raman intensity is 3.3 times larger in CuO nanoflower than in CuO nanograss. The unique CuO nanoflower can be used to optimize the SERS-active platform for detecting the small amount of chemical and biomolecules as a non-destructive method within a short analysis time [33–35].

## Conclusions

Hierarchical nanostructures are investigated for self-cleaning and surface plasmonic applications. We have fabricated the hierarchical nanostructures using one-step oxidation process by controlling the formation of CuO nanoflowers on CuO nanograss. This nanoscale CuO structures provide the desired roughness to have superhydrophobic characteristics. For the nanoflowers on nanograss, the additional hierarchy with semi-reentrant structure can repel small droplet (~10 nL) of various low surface tension liquids such as glycerol, ethylene glycol, olive oil, and 25 % ethanol (mixed with water). Therefore, the hierarchical nanostructures have non-wettable and self-cleaning properties.

The CuO hierarchical structures also enable plasmonic applications as a SERS substrate. Raman shifts of 4-Mpy are observed on Ag-coated CuO nanoflower and CuO nanograss. Raman scattering is enhanced by the interaction between CuO nanostructures and Ag layer. SERS signals are detected where the nanoscale gaps serve as hot-spots. There are many hot-spots that provide sufficiently narrow gaps among the structures of randomly grown CuO nanograss and CuO nanoflower surface. Especially, owing to the narrowly accumulated crystal plates, the CuO nanoflower has 3.3 times larger SERS intensity than that of CuO nanograss structure.

Fabrication of CuO nanograss based on a solution method is relatively simple and fast; thus, this result potentially provides a path toward cost-effective fabrication of a non-wettable surface for self-maintenance applications and a SERS substrate for sensing applications.

## Additional File

Additional file 1:
**A movie of wetting test on hierarchical nanoflowers on nanograss structures.** (MP4 9294 kb)

## Abbreviations

SERS: Surface-enhanced Raman scattering
4-Mpy: 4-Mercaptopyridine
FOTS: Trichloro(1H,1H,2H,2H-perfluorooctyl)silane
CA: Contact angle
CAH: Contact angle hysteresis
